# Xylanase production by *Thermobacillus xylanilyticus* is impaired by population diversification but can be mitigated based on the management of cheating behavior

**DOI:** 10.1186/s12934-022-01762-z

**Published:** 2022-03-15

**Authors:** Romain Bouchat, Frédéric Vélard, Sandra Audonnet, Damien Rioult, Frank Delvigne, Caroline Rémond, Harivony Rakotoarivonina

**Affiliations:** 1 INRAE, FARE, UMR A 614, Chaire AFERE, Université de Reims Champagne Ardenne, 51097 Reims, France; 2 Laboratory of Microbial Processes and Interactions, TERRA Teaching and Research Centre, Gembloux Agro-Bio Tech, University of Liege, Avenue de la Faculté 2B, B140, 5030 Gembloux, Belgium; 3 BIOS EA 4691 “Biomatériaux et Inflammation en site osseux”, Université de Reims Champagne Ardenne, 51097 Reims, France; 4 URCACyt, Flow Cytometry Technical Platform, Université de Reims Champagne-Ardenne, 51096 Reims, France; 5 Plateau Technique Mobile de Cytométrie Environnementale MOBICYTE, Université de Reims Champagne-Ardenne, 51097 Reims, France

**Keywords:** Hemicellulases, Xylanases, *Thermobacillus xylanilyticus*, Successive cultivation, Population diversification, Cell sorting, Substrate switch

## Abstract

**Background:**

The microbial production of hemicellulasic cocktails is still a challenge for the biorefineries sector and agro-waste valorization. In this work, the production of hemicellulolytic enzymes by *Thermobacillus xylanilyticus* has been considered. This microorganism is of interest since it is able to produce an original set of thermostable hemicellulolytic enzymes, notably a xylanase GH11, Tx-xyn11. However, cell-to-cell heterogeneity impairs the production capability of the whole microbial population.

**Results:**

Sequential cultivations of the strain on xylan as a carbon source has been considered in order to highlight and better understand this cell-to-cell heterogeneity. Successive cultivations pointed out a fast decrease of xylanase activity (loss of ~ 75%) and Tx-xyn11 gene expression after 23.5 generations. During serial cultivations on xylan, flow cytometry analyses pointed out that two subpopulations, differing at their light-scattering properties, were present. An increase of the recurrence of the subpopulation exhibiting low forward scatter (FSC) signal was correlated with a progressive loss of xylanase activity over several generations. Cell sorting and direct observation of the sorted subpopulations revealed that the low-FSC subpopulation was not sporulating, whereas the high-FSC subpopulation contained cells at the onset of the sporulation stage. The subpopulation differences (growth and xylanase activity) were assessed during independent growth. The low-FSC subpopulation exhibited a lag phase of 10 h of cultivation (and xylanase activities from 0.15 ± 0.21 to 3.89 ± 0.14 IU/mL along the cultivation) and the high-FSC subpopulation exhibited a lag phase of 5 h (and xylanase activities from 0.52 ± 0.00 to 4.43 ± 0.61 over subcultivations). Serial cultivations on glucose, followed by a switch to xylan led to a ~ 1.5-fold to ~ 15-fold improvement of xylanase activity, suggesting that alternating cultivation conditions could lead to an efficient population management strategy for the production of xylanase.

**Conclusions:**

Taken altogether, the data from this study point out that a cheating behavior is responsible for the progressive reduction in xylanase activity during serial cultivations of *T. xylanilyticus*. Alternating cultivation conditions between glucose and xylan could be used as an efficient strategy for promoting population stability and higher enzymatic productivity from this bacterium.

**Supplementary Information:**

The online version contains supplementary material available at 10.1186/s12934-022-01762-z.

## Background

The use of lignocellulosic biomass has been identified as a promising approach in the biorefinery field for the production of bioderived products like energy, fuels, chemicals and materials to substitute the fossil carbon sources causing global warming [1–3]. In this valorization, hemicelluloses offer new possibilities of industrial applications [4]. These ones are heteropolysaccharides and the most abundant are xylans formed by linear chains of d-xylopyranose linked by β-(1-4) glycosidic bonds [5, 6]. Some substitutes (depending on the plant origin) such as l-arabinofuranose, d-glucuronic and 4-*O*-methyl-d-glucuronic acid and acetyl groups can be linked to the main chains by various bonds. In graminaceaous plant cell walls, the l-arabinofuranose residues can be esterified by phenolic compounds such as ferulic and *p*-coumaric acids which can interfere with the hydrolysis process but also present interesting applications [5–7]. Among the different processes that could be used for lignocellulose depolymerization, the use of enzymes from different sources is a very interesting alternative already reviewed [8–11]. In the context of this work, the production of hemicelluloses by the bacterium *Thermobacillus xylanilyticus* will be considered.

This bacterium is indeed a very promising natural producer of enzymes for the deconstruction of xylans [12]. This bacterium was originally isolated from a farm soil located under a manure heap in northern France) is aerobic, gram-positive, thermophilic and hemicellulolytic [13]. The hemicellulolytic enzymatic arsenal of this bacterium is composed by several enzymes including characterized two xylanases (GH10 and GH11 families, EC.3.2.1.8), one arabinofuranosidase (EC.3.2.1.55) and one feruloyl esterase (EC 3.1.1.73) [14–16]. This bacterium is able to mobilize a complex enzymatic arsenal according to the lignocellulosic biomass composition used as carbon source for promoting microbial growth [17]. The main hemicellulolytic activity of the strain is the GH11 xylanase secreted in the extracellular medium, Tx-xyn11 but it also produces debranching and exoenzymes (such as esterase, xylosidase, and arabinosidase) activities mainly detected in the intracellular compartment [12, 17]. One important feature of the enzymes produced by *T. xylanilyticus* is that they are thermostable and active over a wide range of pH values i.e., from 5 to 8.5 as pointed out by previous studies involving enzymes obtained from cultivation on wheat bran and wheat straw [15, 16, 18] allowing their use under extreme reaction conditions e.g., for pulp and paper processing at high T° and high pH).

However, taking the microbial population out of its natural context for the industrial production of enzymes is still a challenge. Indeed, enzyme production is a highly regulated process, strongly dependent on the extracellular conditions. Additionally, it is known that the release of these enzymes in the extracellular environment can lead to cheating behavior [19]. Cheaters are individuals among the population that benefit from the public goods released by the cooperators (i.e., in *T. xylanilyticus* case, microbial cells that are investing in enzyme production for the release of assimilable carbon sources), but that do not share the cost associated with the release of these public goods [20]. This is exactly what has been observed in this study, upon serial cultivations of *T. xylanilyticus* on xylan.

Typically, these cell-to-cell heterogeneities are due to epigenetic mechanisms leading to fluctuations at the level of the biochemical reactions of the cells, and modulating gene expression [21, 22]. In some case, these population heterogeneities are reinforced by fitness advantage (i.e., in this study, benefiting from the release of assimilable sugars without investing in enzyme production) and can cause a decrease of global productivity due to the presence of some specific non-producer (or low-producer) subpopulations [22, 23].

For addressing such population heterogeneities, high throughput single-cell analyses can be provided by flow cytometry (FC). In this work, two basic FC signals will be mainly used i.e., the forward and side scatter signals (FSC and SSC), accounting for cell size and cell internal structure [24]. This approach can be complemented based on the utilization of metabolic sensors such as Redox SensorGreen (RSG) that allows to discriminate subpopulations in function of their metabolic activities [25].

The production of specific hemicellulose enzymatic cocktails is a key for improving the profitability of the lignocelluloses biorefineries by using all parts of the lignocellulose [3, 26]. *Thermobacillus xylanilyticus* represents an interesting source of thermostable and robust hemicellulolytic enzymes. A bottleneck for the utilization of this strain is the low enzyme production and the decrease of secreted xylanase activity with thime and over successive cultivations. Accordingly, the main goals of this study are then (1) to study and understand the dynamics of enzyme production by population of *T. xylanilyticus*; (2) to attempt improving the enzyme productions by the bacteria. For this latter, whereas some cheater control strategies have been suggested [27, 28], no real implementation of control/mitigation strategies have been considered so far. In this work, a simple and efficient control strategy will be implemented based on alternative cultivation conditions on glucose and xylan. To date, this work is the first to study the subpopulations from an isogenic population of a hemicellulolytic bacterium and their impacts on the extracellular xylanase/enzymatic production. It is also the first work to implement a control of the diversification process by switching between carbon sources during serial cultivations for the production of xylanase by a thermophilic bacterium.

## Results

### Successive cultivations of *T. xylanilyticus* on xylan leads to the progressive decline in xylanase activity

In order to characterize the behavior of *T. xylanilyticus* in presence of xylan and the resulting xylanase production, the growth rate (h^−1^) and its enzymatic activity (IU/mg) were measured during 107.7 generations. For this purpose, successive cultivation in sealed bottles was considered even if the strain is aerobe. Indeed, in sealed bottles, carbon dioxide is added in the headspaces of the bottles. The carbon dioxide and the bicarbonate present in the medium are in equilibrium and create a buffer system with a pH at 7.75 necessary for the growth of the strain as described by Touzel [13]. It is with these conditions that production heterogeneity was observed. These conditions were then necessary to identify the factors influencing the enzymatic production of the strain along the generations.

**Fig. 1:**
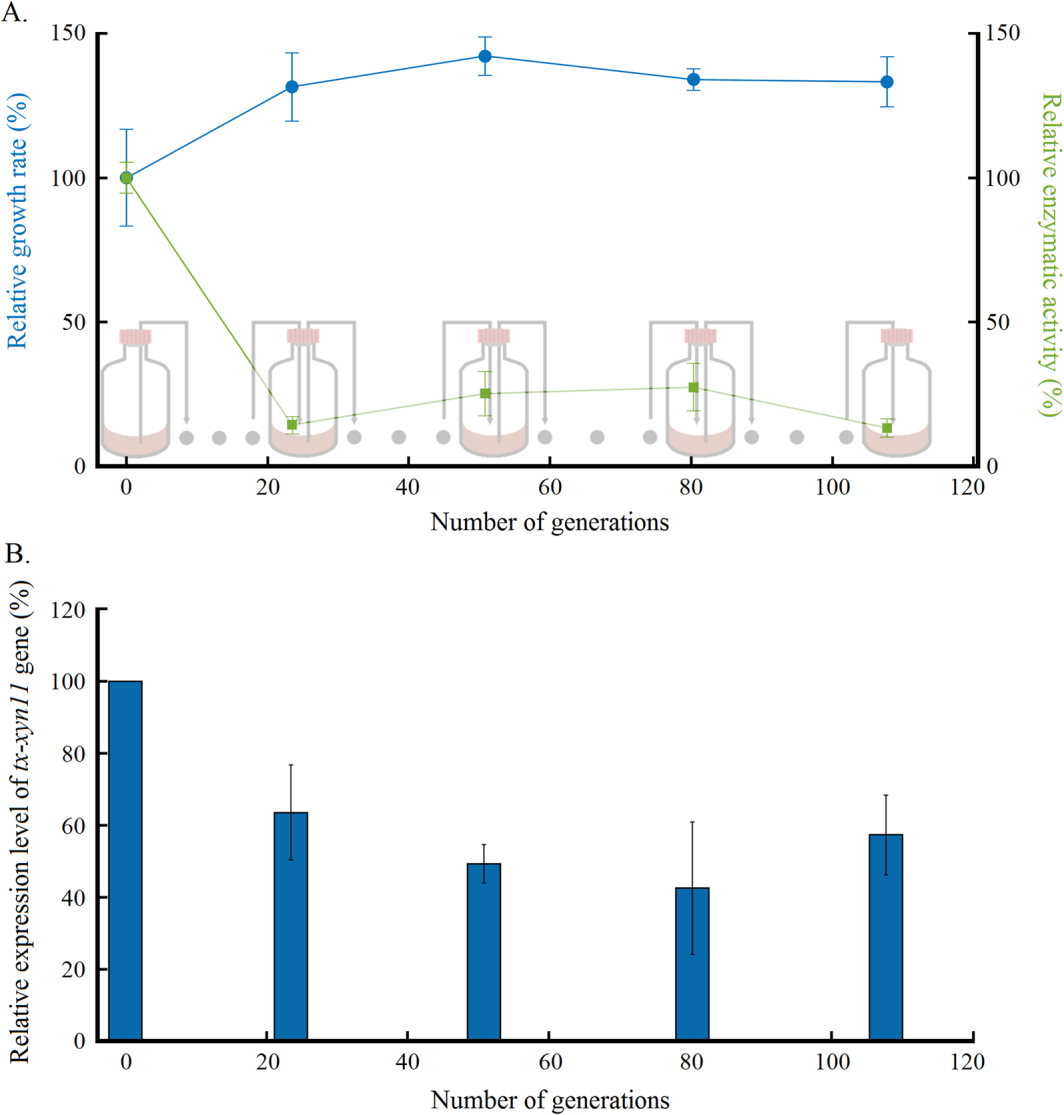
Evolutions of growth rate and xylanase production characteristics of *Thermobacillus xylanilyticus* over generations. Evolution of relative growth rate (blue circles) and relative xylanase activity (green square) over generations normalized with the values at generation 0 (**A**); relative expression level of *tx-*xyn11 gene over generations (**B**). Absolute values of growth rate and enzymatic activity can be found in Additional file 2: Table S1. The level of *tx-xyn11* gene expression was normalized with the 16 S rRNA gene expression before comparison along the generations during successive cultivations with xylan. The first cultivation (generation 0) was then defined as the 100% expression level and as reference sample. The expression levels over the generations were expressed as the fold increase of *tx-xyn11* mRNA level over the generation 0

The initial growth rate on xylan for the first cultivation was 0.72 ± 0.12 h^−1^ (generation 0, Additional file 2: Table S1), and this value was considered as the reference (100%, generation 0, Fig. 1). We observed that the growth increased over the generations, this increase being more important and significant (p < 0.01) during the first generations (increase of 31.56% after 23.5 generations). A stabilization was then noticed around a value of 0.95 h^−1^ until the end of the successive cultivation experiment. During this stabilization, no significant difference was observed. The xylanase activity, the main enzyme secreted by *T. xylanilyticus*, was also followed. At the beginning of the cultivations, xylanase activity was 141.36 ± 7.76 IU/mg of proteins. The xylanase activities revealed a drastic decrease over generations, a significant decrease of almost 88.86% during the first 23.5 generations (p < 0.01) followed by a stabilization around a value of 28.10 ± 10.32 IU/mg (Additional file 2: Table S1). At the end of the successive cultivations (after more than 100 generations), the xylanase activity value represented only 18.51 ± 4.54 IU/mg i.e., exhibiting a significant reduction of more than 90% by comparison with the first cultivation (p < 0.01).

The evolution of the *tx-xyn11* gene expression, encoding the main xylanase GH11 secreted by *T. xylanilyticus* was also assessed along generations during cultivations on xylan and on glucose as a control. At the beginning of the cultivations, and as expected, the expression level of *tx-xyn11* gene was very low in presence of glucose. On xylan, *tx- xyn11* expression level was almost 30-fold higher than on glucose (Additional file 1: Fig. S1), this observation being in accordance with the induction level previously recorded for this strain [15].

Upon successive cultivations on xylan, a progressive decrease of *tx-xyn11* expression over the generations was observed (Fig. 1B). This decrease accounted for 36.49% of the maximum level after 23.5 generations by comparison with the *tx-xyn11* expression level measured at the beginning of the cultivation tests which was significant (p < 0.01). After this first decrease, the expression level of *tx-xyn11* remained close to 60% of the initial level for 108 generations. Interestingly, the evolution of the expression level followed the same trend as the one recorded for the enzymatic activity during successive cultivations on xylan (Fig. 1A). Gene expression can be thus considered as the main cause for the decrease observed for the xylanase activity. Considering the fact that the xylanase is an extracellular enzyme and that some cells can adopt a cheating strategy for avoiding the cost related to the synthesis of this molecule [29], single-cell experiments were then considered for determining the possible impact of cell-to-cell variability on xylanase production.

### Flow cytometry (FC) reveals two subpopulations exhibiting different light scattering properties

**Fig. 2:**
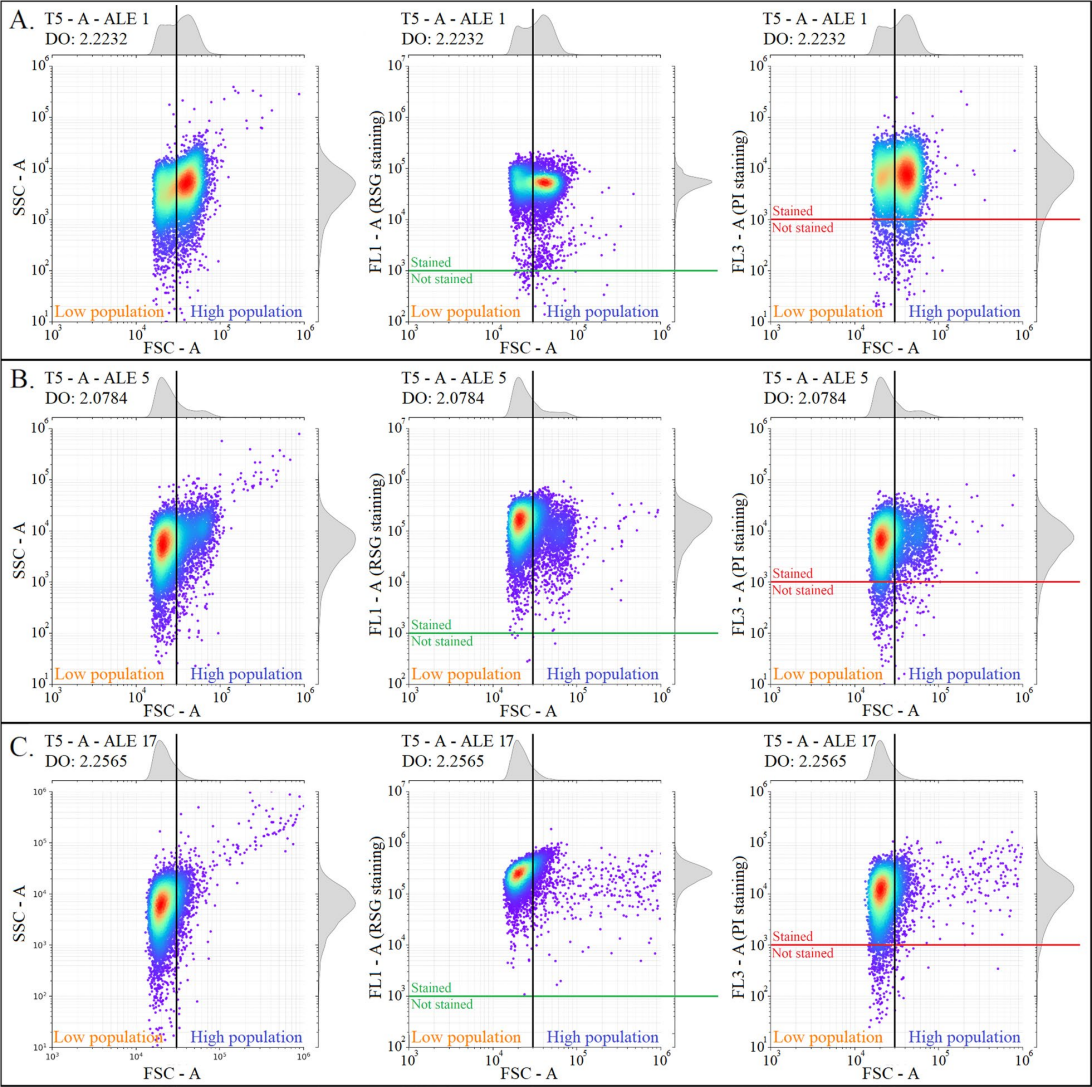
Subpopulations of *Thermobacillus xylanilyticus* analyzed by flow cytometry during successive cultivations on xylan. The subpopulations were resolved based on the FSC-A values. The segregation between the two subpopulations was done by considering a gating value of 30,000 in FSC-A (orange line). The figures represent the SSC-A values (on the left), the FL1-A values (on the middle) and the FL3-A values (on the right) in function of the FSC-A values. Generations 0 (**A**); 23.5 (**B**) and 107.7 (**C**) respectively

FC analyses were then performed for determining the possible occurrence of different subpopulations of *T. xylanilyticus* during the successive cultivation experiments. Results highlighted the presence of two subpopulations in *T. xylanilyticus* cultivations after 5 h of cultivations (Fig. 2A–C). Indeed, the segregation between the two subpopulations can be observed based on the forward scatter (FSC) signal which is proportional to the cell size. A FSC-A threshold value of 30,000 (Arbitrary Unit) can discriminate two subpopulations. The subpopulation with values below 30,000 in FSC-A has been called *low* subpopulation (*L*) and the subpopulation with values above 30,000 in FSC-A has been called *high* subpopulation (*H*).

**Fig. 3:**
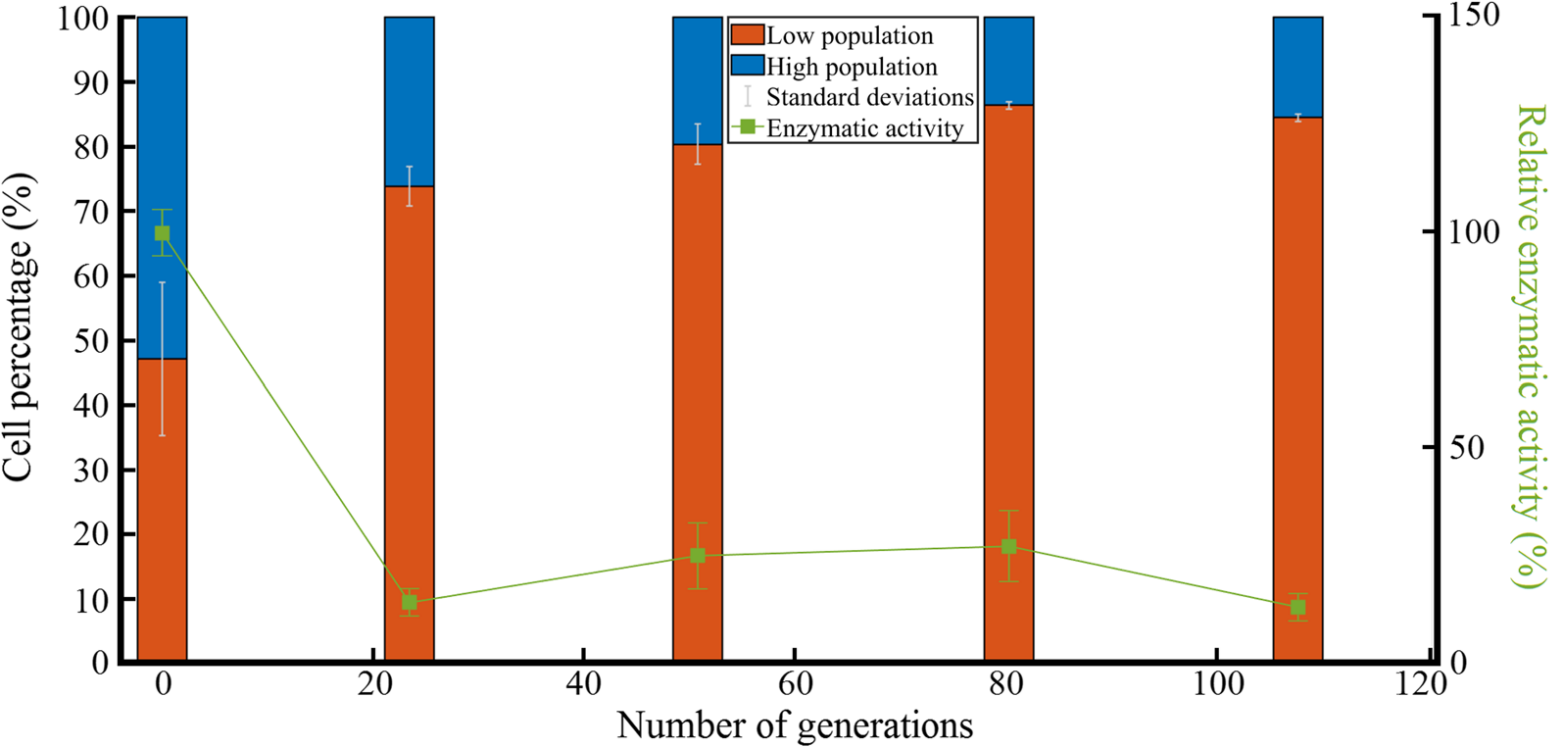
Evolution of cell percentage and xylanase activity for each subpopulation upon successive xylan cultivations. The orange and blue bars represent the means of cells belonging to the *low* and *high* subpopulation respectively. The standard deviations are represented by white lines. The evolution of the enzymatic activity expressed in IU/mg of proteins (green lines) is also presented

This clear segregation based on the FSC-A threshold allowed tracking the two subpopulations over the different generations (Fig. 3). At the beginning of the cultivations (generation 0), the two subpopulations were present at a similar level (47.09 ± 11.84% and 52.91 ± 11.84% for the *low* and the *high* subpopulation respectively). The evolution of the ratio between the subpopulations over the different generations was marked by an increase of the *low* subpopulation. This increase was more important during the 23.5 first generations, where the percentage rose significantly from 47.08 to 73.87% (p < 0.01) and then stabilized (with no more significant change) to 83.77% after 108 generations.

The two subpopulations did not exhibit any differences upon staining respectively with RedoxSensor green and Propidium Iodide (FL1-A and FL3-A values), indicating that it did not differ by their respective metabolic activity and membrane permeability.

**Fig. 4:**
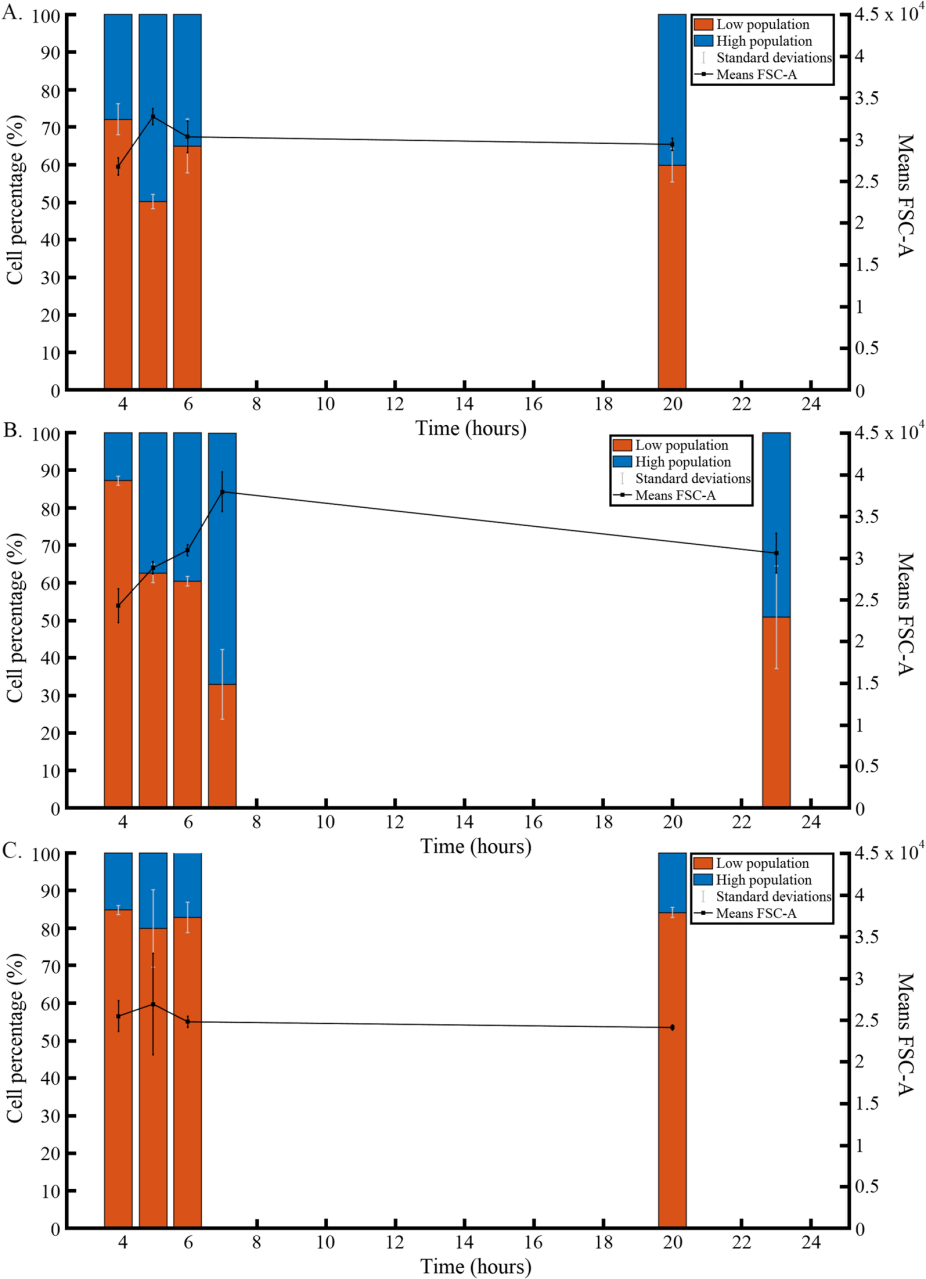
Physiological changes of *T. xylanilyticus* detected by flow cytometry on different substrates. The substrates considered are either xylan (**A**), glucose (**B**) or wheat bran (**C**)

Complementary FC analyses were performed on different substrates (i.e., xylan, glucose and wheat bran) in order to better characterize the occurrence of the two subpopulations (Fig. 4). Wheat bran was selected as an interesting carbon source, considering its high xylan content [17]. At the beginning of the culture and during the exponential phase (4 h of cultivations) whatever the substrate used, the *low* subpopulation was dominant and accounted for 72.1, 87.3 and 84.9% of the total number of cells on xylan, glucose and wheat bran respectively. At the end of exponential phase on glucose and xylan, an increase of the *high* subpopulation was observed (Fig. 4A, B). For wheat bran cultivations, the *low* subpopulation remained dominant during all the experiment. It is also on wheat bran that less sporulation was always observed. Then, the balance between the two subpopulations seems to be easier to achieve when rich carbon source (i.e., glucose) is considered instead of more difficult to assimilate ones (i.e., xylan and wheat bran). Taken altogether, these data point out a possible occurrence of sporulation. This hypothesis is reinforced by the fact that cells triggering the formation of endospore tend to modify their light scattering properties as detected based on flow cytometry analyses. Accordingly, microscopy analyses will be considered in the next section.

### Cell sorting reveals that the high-FSC subpopulations contains cells at the onset of sporulation

**Fig. 5:**
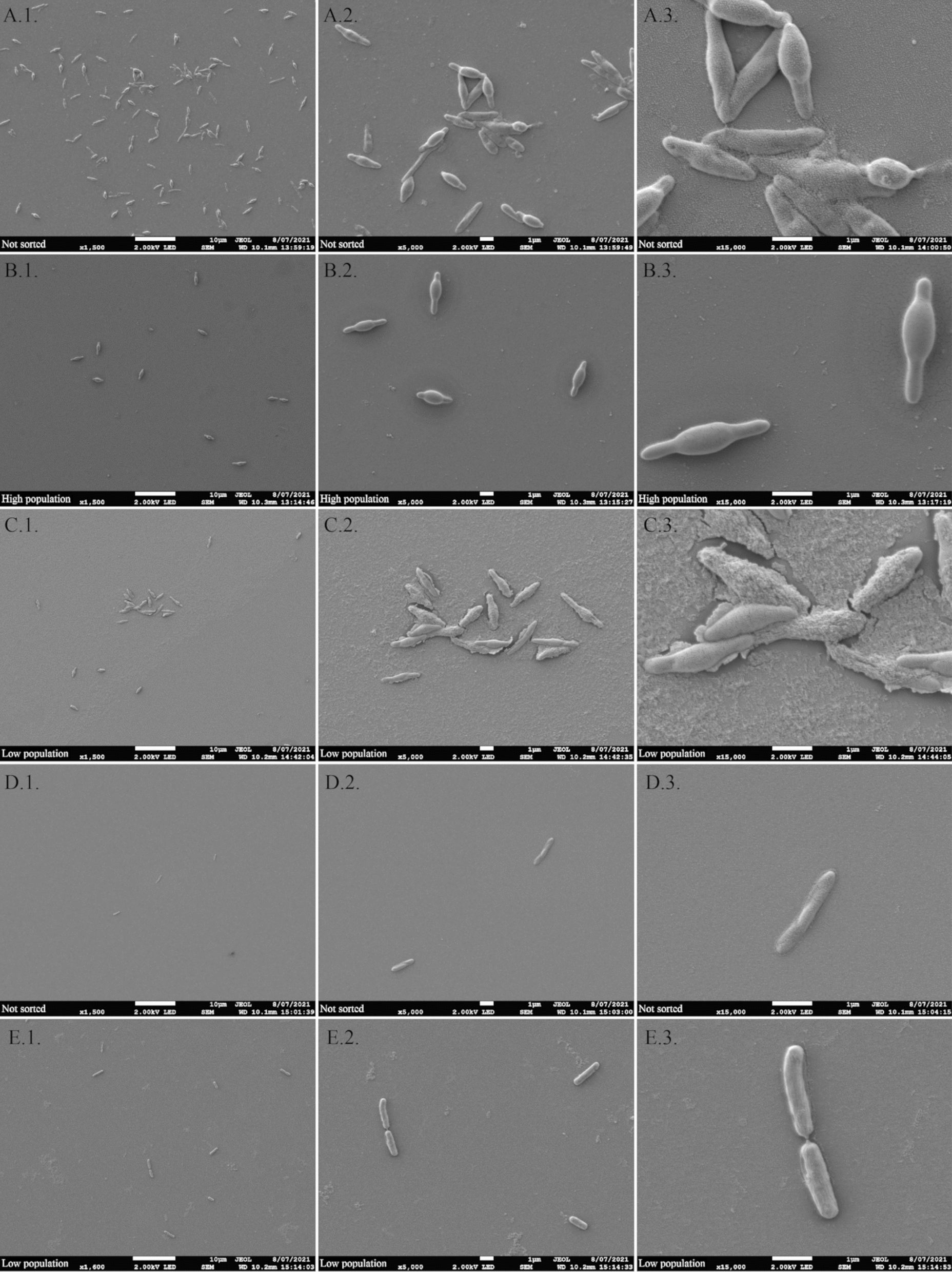
Morphological changes along the generations. Cells were observed by SEM at magnification 1500-fold (1), 5000-fold (2) and 15,000-fold (3). Generation 0 not sorted (**A**), generation 0 sorted to obtain *high* subpopulation (**B**), generation 0 sorted to obtain *low* subpopulation (**C**), G50 not sorted (**D**) and G50 after cell sorting (**E**)

In order to get more insights about the possible physiological differences between the *low* and the *high* subpopulations, cells collected from samples acquired during successive cultivations on xylan were sorted based on their FSC properties, and morphological analyses were performed with Scanning Electron Microscopy (SEM). More specifically, the generations for which a strong modification of the enzyme activity was recorded were analyzed i.e., the 20th generation (G20) and the 50th generation (G50). The SEM pictures revealed that two types of morphologies were present at G0 after 5 h of cultivation (Fig. 5A) i.e., either very long (or dividing) cells and sporulating cells. After cell sorting of the two subpopulations at G0, the *high* subpopulation contained cells with well-formed sporangia in central position (Fig. 5B). Some sporulating cells were also observed in *low* subpopulation but at an earlier sporulation stage (Fig. 5C). From G20 to G50, a predominance of the *low* subpopulation was observed by flow cytometry, most of the cells being in the vegetative state (at the same cultivation time) up to G50 (Fig. 5D). The cell sorting at G20 and G50 revealed the predominance of the *low* subpopulation with vegetative cells (Fig. 5E).

These observations confirmed the morphological and physiological diversification of the subpopulations upon successive cultivations and the progressive decrease of the *high* subpopulation. It also correlated the *high* subpopulation with cells exhibiting advanced sporulation stage and the *low* subpopulation containing vegetative cells.

**Fig. 6:**
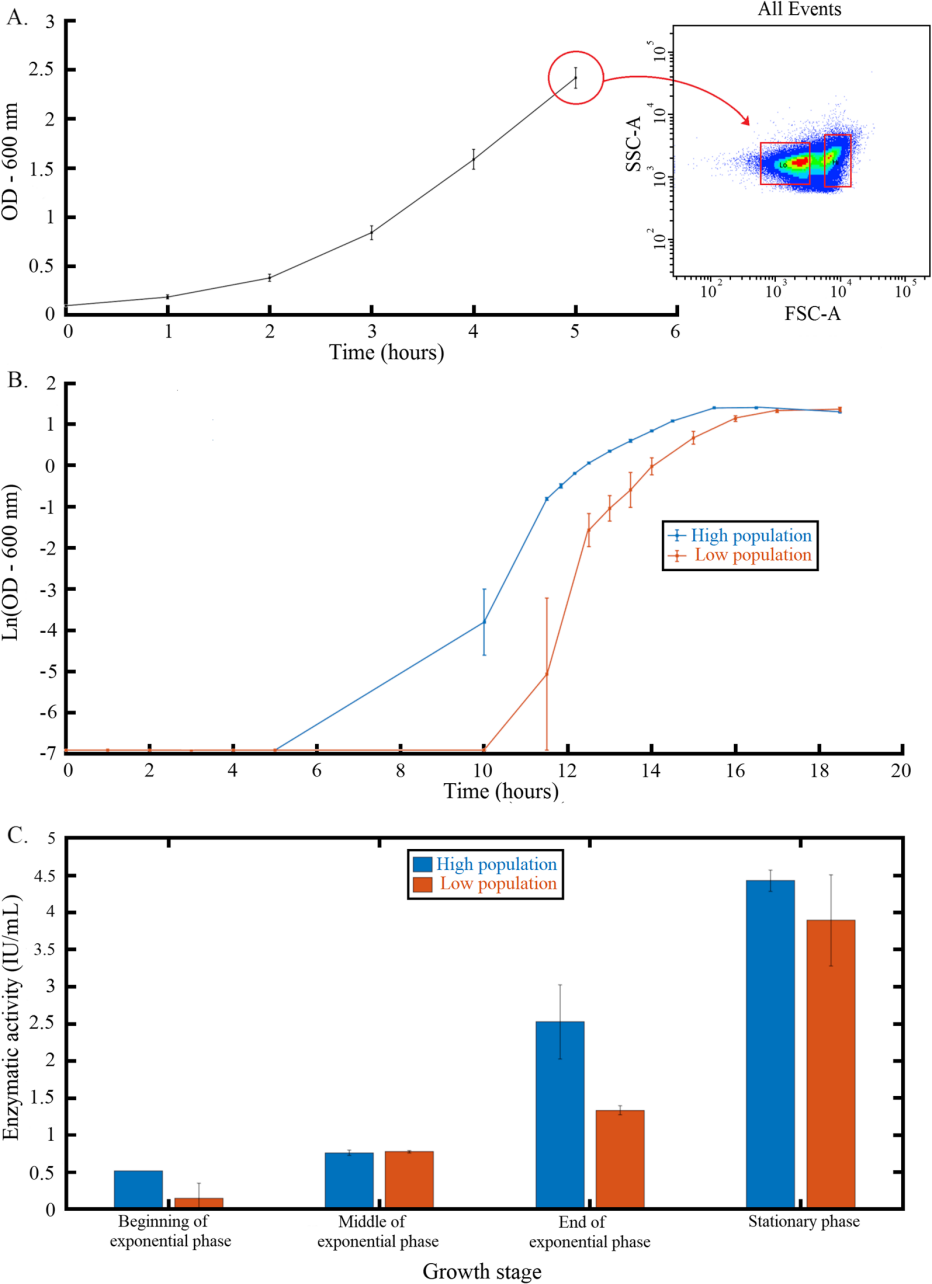
Characterization of the two subpopulations of *T. xylanilyticus*. Growth and flow cytometry profiles of generation 0 cultivations before cell sorting (**A**). The red boxes on the FSC-SSC dotplot represent the gates used for discriminating the two subpopulations; growth profiles of the two subpopulations (**B**); xylanase activities for the two subpopulations at different growth phases (**C**). The blue lines and bars represent the *high* subpopulation, and the orange ones represent the *low* subpopulation

In order to determine the impact of this diversification process on the production of extracellular xylanase by *T. xylanilyticus*, the *low* and the *high* subpopulations were sorted and subcultivated. The cell sorting was done at generation 0, after 5 h of cultivation during the exponential growth phase (Fig. 6A) when the two subpopulations were present similarly at approximately the same ratio.

The growth rate and the xylanase activity for each subpopulation were assessed in the presence of xylan. A common feature observed for the two subpopulations was the lag phase, which was very long, probably due to the very low inoculum used after cell sorting. However, the lag phase was longer for the *low* subpopulation (Fig. 6B). The growth began after 5 h for the *high* subpopulation and after 10 h of incubation for the *low* subpopulation. However, the duration of exponential growth phase was similar for the two subpopulations. (3, 5 and 4 h for the *low* and *high* subpopulation respectively) with growth rates of 0.55 ± 0.01 h^−1^ and 0.82 ± 0.02 h^−1^ respectively.

About the xylanase activity, an important difference of xylanase activity was noticed at the beginning and at the onset of the exponential phase. Statistical analyses did not allow to conclude to a significant difference (p = 0.127) but the means xylanase activity production was 3.5-fold higher for the *high* subpopulation by comparison with the production level of the *low* subpopulation at the beginning of the exponential phase (~ 0.52 ± 0.00 IU/mL vs. ~ 0.15 ± 0.21 IU/mL for the *high* and *low* subpopulations respectively) (Fig. 6C). At the end of the exponential phase, a difference between the two subpopulations was also observed as the xylanase activity produced by the *high* subpopulation was 1.89-fold higher than the one observed for the *low* subpopulation. It was not a significant difference, but it was more important than at the onset of sporulation (p = 0.0793). A sharp increase of xylanase production was observed between the end of exponential phase and stationary phase (1.33 ± 0.5 IU/mL to 3.89 ± 0.14 IU/mL and 2.52 ± 0.06 IU/mL to 4.43 ± 0.61 IU/mL for the *low* and *high* subpopulations respectively) with the two subpopulations exhibiting similar level of xylanase concentration after this phase. The cell sorting confirmed then the presence of different subpopulations related to xylanase production. The difference is more noticeable with the required time for the production and not the production level itself. Indeed, both populations exposed xylanase production, but it required more time for the low population which needs to adapt its metabolism for production when cultivated alone.

### Improving the xylanase production based on a cheater management strategy

**Fig. 7:**
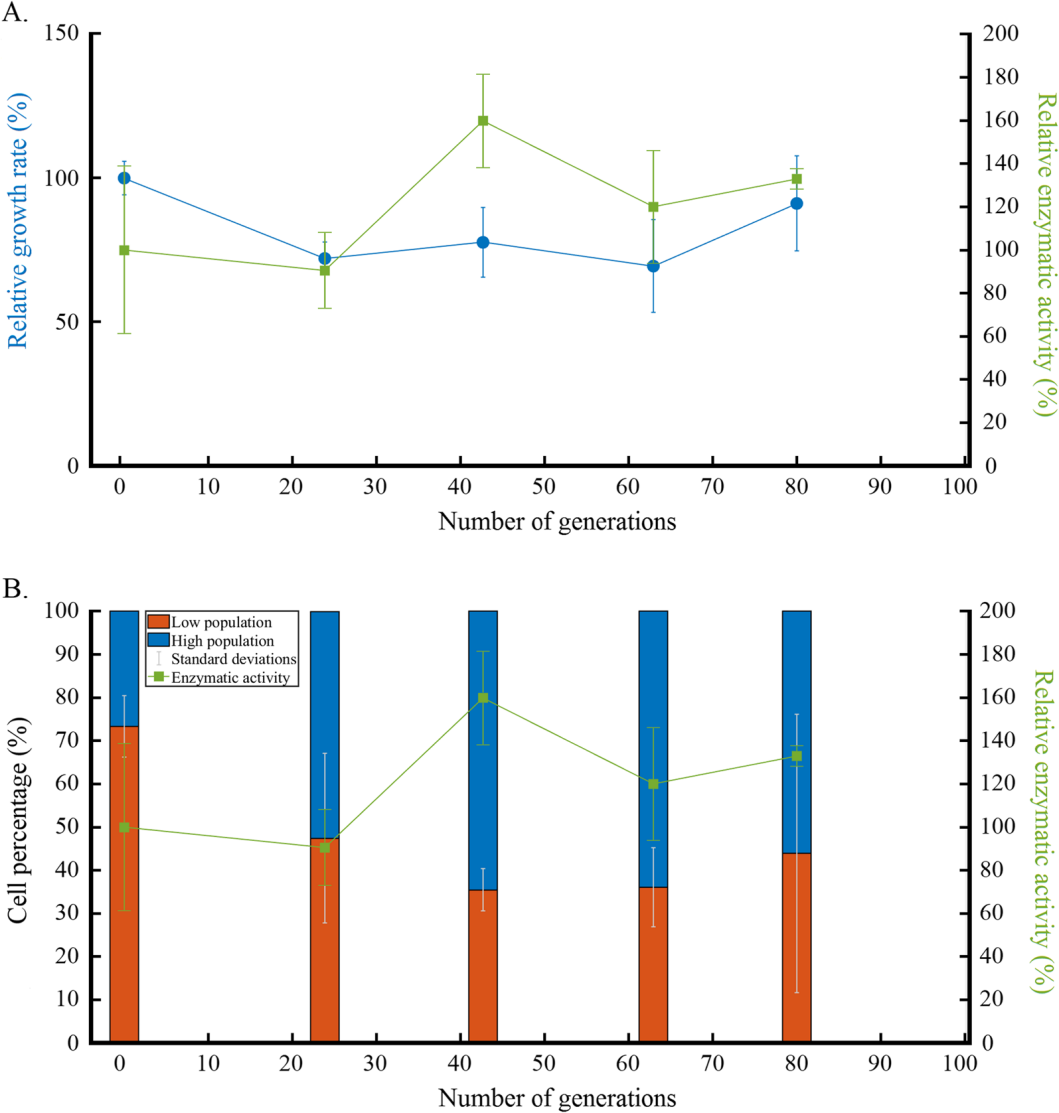
*Thermobacillus xylanilyticus* subpopulations evolution during glucose successive cultivations with xylanase activity being recorded after xylan switching. Relative evolution of the growth rate (blue circles) and enzymatic activity after substrate switch (green squares) over generation (**A**); evolution of cell subpopulations in percentage over generations after flow cytometry analyses (**B**). The xylanase activity was measured after a switch of carbon source from glucose to xylan at the same growth stage (green lines in **A** and **B**)

As shown in the previous section, the presence of a subpopulation exhibiting reduced extracellular enzyme production during successive cultivations led to a global decrease of xylanase production by *T. xylanilyticus*. This *low*, non-producing, subpopulation was considered as a cluster of cheater cells. In order to validate this hypothesis, and as an attempt to control the occurrence of cheater cells, successive cultivations on glucose instead of xylan were considered. Indeed, glucose being a more easily usable carbon source, *T. xylanilyticus* does not require xylanase for its growth with this carbon source. The strategy was then to prevent the selection of one subpopulation along generations by providing an important pool of usable carbon source before switching to more complex substrates requiring enzymes to produce a usable form of carbon source. For this, *T. xylanilyticus* was cultivated during the successive cultivations experiment on glucose and carbon source shifts from glucose to xylan were done to evaluate the impact on xylanase production. The occurrence of the two subpopulations was done on flow cytometry analyses and xylanase production was also assessed (Additional file 3: Table S2 and Fig. 7).

The growth rate on glucose at the beginning of the cultivations (generation 0) was 0.87 ± 0.05 h^−1^ and did not change significantly over the generations considering the standard deviations (Fig. 7A).

At the beginning of the cultivation, the measured xylanase activity represented 217.07 ± 84.20 IU/mg of proteins (values closed to the ones obtained at generation 0 on xylan successive cultivations, 141.36 ± 7.77 IU/mg of proteins, considering the standard deviations). The xylanase enzymatic activity after the carbon source switch (from glucose to xylan) over the different generations pointed out a stabilization of the production and the enzymatic levels quantified were comprised between 217.07 ± 84.20 and 288.55 ± 10.70 IU/mg between generations 0 to 79.9 with a peak at 346.99 ± 47.07 IU/mg after 42.7 generations (Fig. 7A). In contrast to the experiments carried out on xylan, no significant loss of activity was observed when xylan was replaced by glucose. The level of xylanase activity was relatively high for all the generations when glucose was used as the main carbon source and subpopulations were switched to xylan at the end of each cultivation cycle. Globally the production of xylanase by *T. xylanilyticus* over successive cultivations was stabilized when glucose was used as the main carbon source (data from Additional file 3: Table S2, by comparison with data from Additional file 2: Table S1).

The flow cytometry analyses performed during the successive cultivations on glucose pointed out the presence of the two previously observed subpopulations (*low* and *high*) such as for the successive cultivations on xylan. Figure 7B showed that, at the beginning of the experiment, the *low* subpopulation represented 73.36 ± 7.13% of the total population. Over successive cultivations on glucose, the percentage of cells into the *high* subpopulation significantly increased up to 64.51% (p < 0.05) after 42.7 generations where the maximal level of xylanase activity was obtained. On the opposite, successive cultivations on xylan led to a drastic reduction of the *high* subpopulation.

## Discussion

Successive cultivation tests pointed out a population degeneration effect at the level of extracellular xylanase activity by *T. xylanilyticus*. This decrease was linked with a significant drop at the level of the transcriptional activity linked to this enzyme and was characterized for the first time for this bacterium. This kind of production decrease has already been observed for many other microorganisms (bacteria and fungi) of industrial interest [30]. The main question is to know whether this decrease can be attributed to a global reduction of enzyme production by all the cells within the population or due to a subpopulation of cells. Single-cell analyses revealed that two subpopulations were generated during the successive cultivations on xylan i.e., a subpopulation exhibiting a *low* FSC signal and the other exhibiting a *high* FSC signal. Such modulation of the FSC signal suggests a significant alteration of the cellular morphology [31]. According to microscopy observations, the *high* subpopulation comprised cells in a premature stage of sporulation (onset to sporulation being triggered during the exponential phase). Upon cell sorting and subcultivation, the *high* subpopulation exhibited a shorter lag phase and an earlier xylanase production, suggesting that these sporulating cells are still able to produce the target enzyme. These differences at the level of the enzyme production capability suggest that a cheating mechanism can be at the origin of the population degeneration effect observed during successive cultivation. The cheater phenotype is prone to appear when microbial population relies on an extracellular enzymatic production to degrade complex substrates into an usable [32, 33]. In *T. xylanilyticus* case, since xylanase is excreted to the extracellular medium, some cells can avoid the metabolic burden associated with the production of this enzyme but still take benefit from the monomers released by the active members of the population [19].

**Fig. 8:**
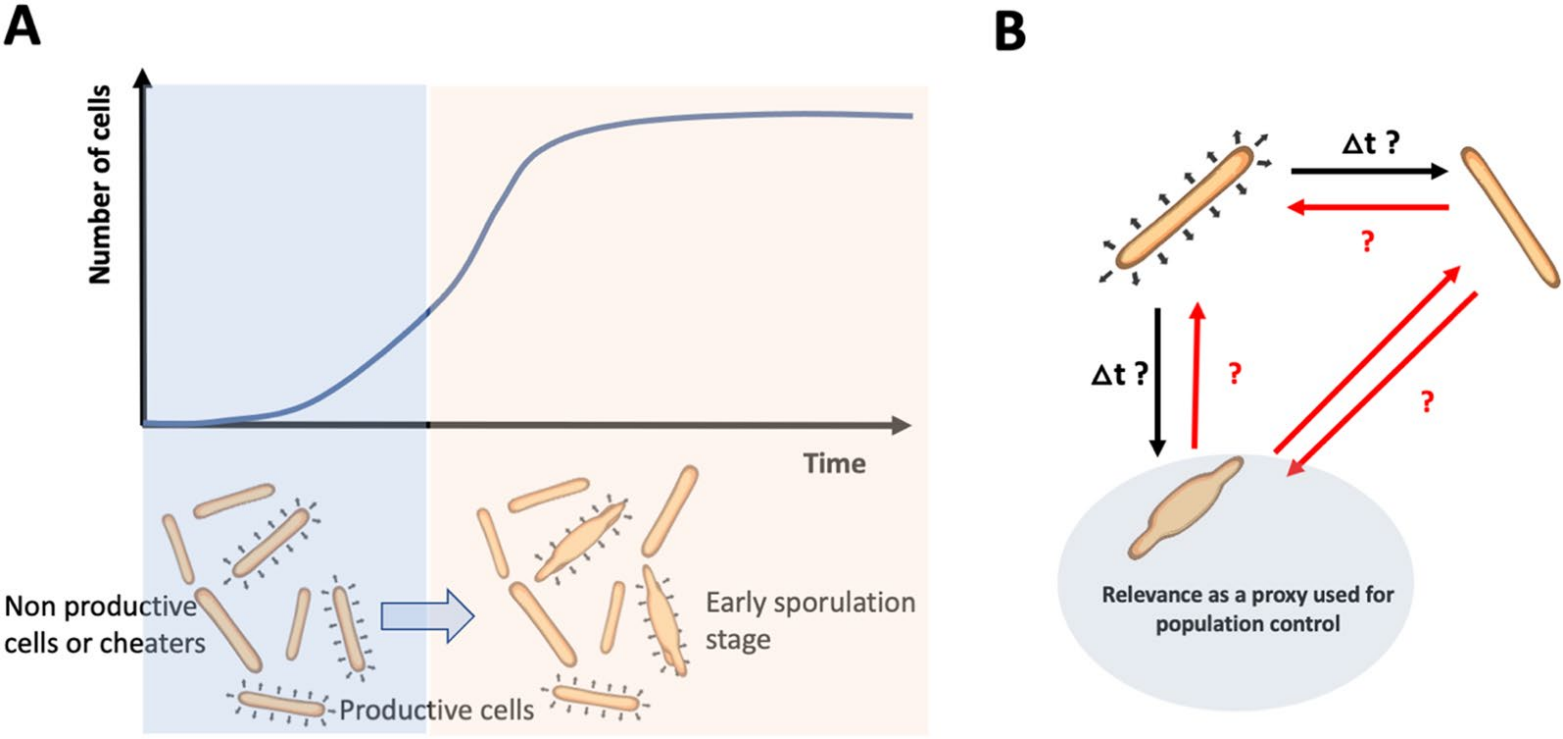
Model for *T. xylanilyticus* diversification at the population (**A**) and single-cell level (**B**)

According to these observations, a preliminary model can be advanced (Fig. 8). At the population level (Fig. 8A), diversification and split into two subpopulations with distinct FSC properties are observed during the exponential phase. In this case, the premature sporulation can be due to the metabolic burden carried by the non-cheating cells. At the single-cell level, the picture is more complex (Fig. 8B). First, if there is a need for a better characterization of the population dynamics, the transition rates between the main phenotypes (either productive or non-productive cells) and morphotypes (either sporulating or non-sporulating cells) need to be quantified. Additionally, a series of unknown remains i.e., some transitions that have not been considered in the initial view of the process (delineated by red arrows on Fig. 8B). However, a common observation is that the “sporulating” state can be easily detected based on single-cell technologies and could be used as a proxy for detecting population degeneration. Some studies have already proven the presence of sporulating cells or morphologically different cells directly impacting the productivity of the process such as *Clostridia* [34, 35]. This work is the first to study bacterial subpopulations from an isogenic global population and highlights their impacts on an hemicellulase production. Some studies already exist for the application of flow cytometry for the identification and cell sorting (by FACS) of fungal cells (*Aspergillus niger* and *Trichoderma reesei*) to detect cellulase activity and select improved strains for cellulase production but not on hemicellulase production. Moreover, this study provides a strategy based on a cheater management which is a first for improving xylan hydrolysis of lignocellulosic biomasses. Unlike fungal studies, it is not based on mutagenesis, screening and cell sorting.

Advanced single-cell technologies could be implemented in order to refine the resolution about microbial population dynamics. Among them, single-cell cultivation microfluidics could be used for tracking cells and determining more precisely the transition rate between the different morphotypes [36]. Another alternative would be to use automated flow cytometry and feedback control. Such technologies have already been used for characterizing population dynamics of Gram-negative bacteria based on an approach called segregostat [37, 38].

## Conclusions

This study pointed out the importance of populations diversification processes during long-time cultivation experiments. Four main observations can be highlighted: (1) the strain degeneration at the level of xylanase production from *T. xylanilyticus* along generations on xylan (decrease of 88.86% after 23.5 generations); (2) the presence of two different populations for this bacterium; (3) the difference between the two populations (the *low* population which is not sporulating but less producing and the *high* population which is sporulating but more producing) and the impact of balancing the two population on the xylanase production (selection of the *low* population along generations with xylanase production decrease); (4) the impact of substrate switching (from glucose to xylan) on populations stability according to xylanase activity (increase of 1.54-fold) and stability of this production with no more decrease of xylanase production along generations. All these elements open up new perspectives for the effective control of population diversification, notably by using advanced single-cell technologies.

This study is the first to date to highlight phenotypic diversification process as being responsible for the progressive decrease in xylanase production by a thermophilic hemicellulolytic bacterium. Also, based on the characterization of the diversification process, a mitigation strategy has been derived in order to force the population for producing the target enzyme. This strategy, relying on the alternance between glucose and xylan, was used effectively to mitigate the diversification of the population based on the FSC signal, leading to an improved xylanase production during successive cultivation. This strategy has been notably applied in adaptive evolution studies by switching between cellobiose and glucose in order to obtain *Thermobifida fusca* strain exhibiting higher cellulase production [39]. In *T. xylanilyticus* case, phenotypic diversification is more likely to occur, and such strategy could be implemented into a more sophisticated control procedure relying on automated single-cell measurement in order to prevent the appearance of cheaters during successive or long-term continuous cultivations.

## Methods

### Strains and media

*Thermobacillus xylanilyticus XE9/11/91* isolated from a farm soil under a manure heap in northern France, already characterized by different studies and conserved in glycerol at − 80 °C was used in this work. The bacterium was cultivated on basal medium composed by three different solutions, a macro-mineral solution, a vitamin solution, and a metallic trace solution complemented with NH_4_Cl (1 g/L), yeast extract (2 g/L), NaHCO_3_ and supplemented with 10% CO_2_ as previously described by [13]. Cultivation volumes are 10 mL or 50 mL of media in sealed contents (100 mL or 500 mL bottles). Various carbon sources were used: xylan from beechwood 5 g/L (Roth), glucose 5 g/L (Sigma Aldrich) or destarched wheat bran 10 g/L (ARD Pomacle-Bazancourt, France).

### Successive cultivations of *Thermobacillus xylanilyticus*

**Fig. 9:**
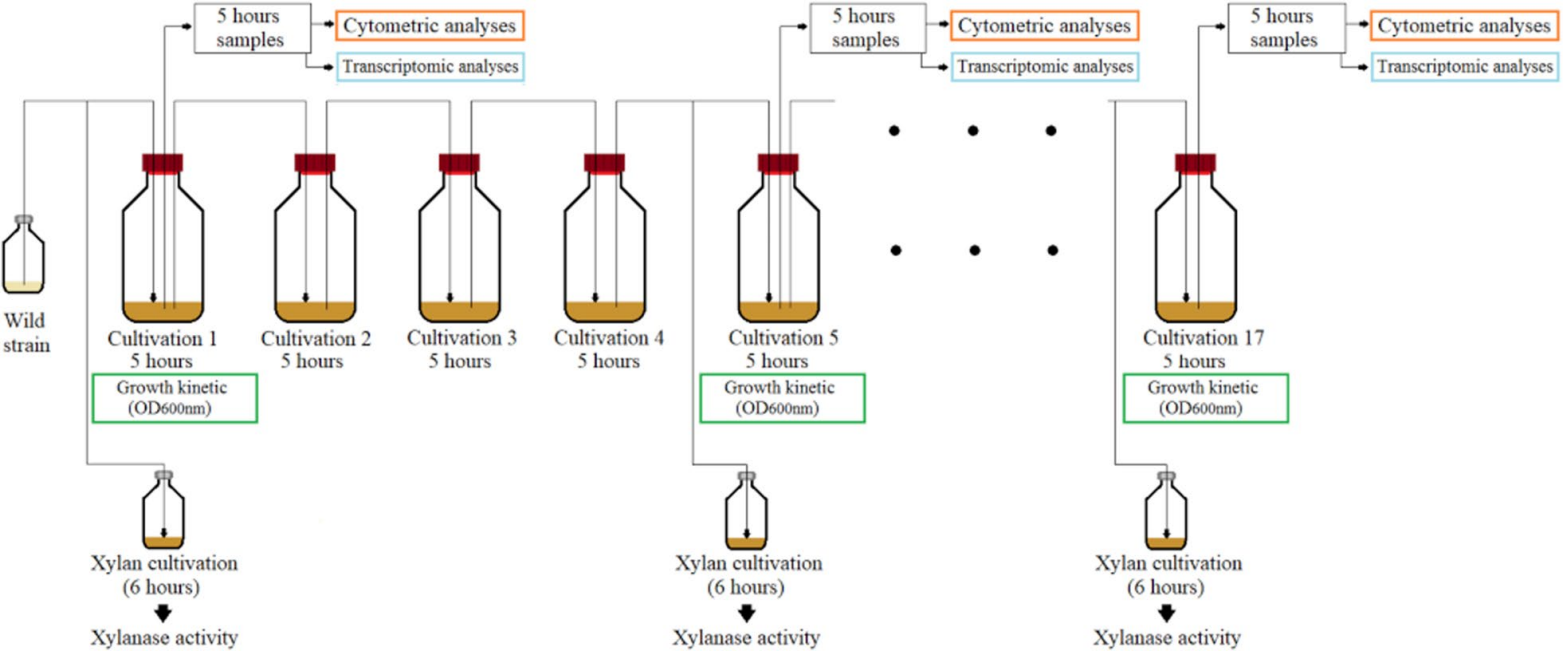
Schematic representation of the successive cultivations experiment of *Thermobacillus xylanilyticus*

The strain was regenerated from glycerol stock and an overnight non sporulated preculture (OD_600 nm_ between 1.5 and 2) was prepared on glucose basal medium at 50 °C and 150 RPM in glucose basal medium with Multitron shakers (Infors). The xylan media (in 50 mL volume) were inoculated with the preculture to reach a start optical density of 0.1 for the cultivations start. The cultivation was done at 50 °C, 150 RPM during 5 h in xylan basal medium. After 5 h, a new cultivation was started from the previous one. The successive cultivation was performed every 5 h (Fig. 9). Each 4 cultivations (G20 or generation 23.5, G50 or generation 50.8, G80 or generation 80.3 and G100 or generation 107.7 for xylan), a following of the growth was done by measuring the OD_600 nm_. The growth rate was calculated during the exponential phase with the formula: µ_max_ = ln (N_2_/N_1_)/t_2 _− t_1_ with N representing the bacterial population (here the optical density measured) and t the cultivation time. The generation numbers (n) were directly calculated with the formula: n = T/g where g represents the generation time (g = ln2/µ_max_) and T the total cultivation time. Successive cultivations experiment was performed in triplicate for 17 serial transfers (corresponding to more than 100 generations).

For the followed generations, population analyses were also done by cytometry, xylanase activity production and the xylanase (*tx-xyn11*) gene expression measured. Figure 9 summarizes the main experiments performed during the successive cultivations.

### Measurements of the xylanase enzymatic activity

To evaluate the xylanase activity secreted by *T. xylanilyticus*, for each followed generation, new 6 h (to reach the early stationary phase) cultivations on xylan in 10 mL were prepared. At the end of the cultivations, samples were centrifuged at 3354×*g* for 10 min (Sorvall ST 8R centrifuge, Thermo Fisher Scientific) at 4 °C and the supernatant was recovered. The xylanase activity was determined in triplicate by using the reducing end sugars measurement according to the procedure described by [40].

Briefly, 0.1 mL of the supernatants (diluted or not) are incubated in 0.9 mL beechwood xylan (Roth) at 0.5% w/v homogeneously suspended in 50 mM sodium acetate buffer (pH of 5.8) at 60 °C for 10 min. The reducing sugars were measured by following the absorbance at 420 nm on a Specord 200 Plus uv/vis spectrophotometer (Analytik Jena) and by comparing the values with a standard curve done with varying concentrations of xylose. The activity was expressed in IU/mL. One international unit (IU) of enzyme activity was defined as the quantity of enzyme (xylanase) required to liberate one µmol of equivalent xylose per minute at 60 °C.

The IU values were normalized with the quantity (in mg) of total protein in the supernatants. The concentrations of total protein in the samples were determined by the Bradford procedure [41] with a commercial reagent 5× (Serva) as recommended by the supplier. After normalization, the activity was expressed in IU by milligrams of total proteins (IU/mg).

### Measurements of the *tx-xyn11* gene expression

The cultivation samples for gene expression (5 mL) were centrifuged at 3354×*g* for 10 min (Sorvall ST 8R centrifuge, Thermo Fisher Scientific) at 4 °C. Cell pellets were kept in 1.5 mL of RNAlater at − 80 °C before the analyses. The cells pellets were used for RNA extraction with RNeasy Mini Kit (Qiagen) by following the supplier recommendations. The obtained RNA solutions were treated with RNase-Free DNase Set (Qiagen) to prevent the presence of DNA in the samples. Absence of DNA was verified by polymerase-chain reaction with DreamTaq™ Hot Start Green PCR Master Mix (Thermo Fisher Scientific) by using specific primers for *tx-xyn11* gene, followed by electrophoresis migrations on RNase-free TBE buffer 1× agarose gel 1% (Mupid® One Electrophoresis System, Eurogentec). The RNA concentrations were determined using a Qubit™ Fluorometer and Qubit® RNA Assay Kit (Invitrogen).

First strand cDNAs were synthesized by using SuperScript® IV First-Strand cDNA Synthesis Reaction kit (Thermo Fisher Scientific) with 500 ng of total RNA in presence of random hexamer primers (50 µM), DNTP mix (0.5 mM of each), 1× SuperScript IV buffer, DTT (100 mM), RNAseOUT™ Recombinant RNase Inhibitor (2 U/µL) and 800 U (or 40 U/µL) of Superscript IV reverse transcriptase in a final volume of 20 µL. The synthesis was done at 23 °C for 10 min followed by a step at 52.5 °C for 10 min. An inactivation was done at 80 °C for 10 min. The generated cDNAs were kept at − 80 °C until utilization.

Quantitative PCR was realized with a QuantStudio™ 3 Real-Time PCR System (Applied Biosystems™) to determine the *tx-xyn11* gene expression. Specific primers (forward primer: GACGGCACGCAGACGTTCCA, reverse primer: GCCTTCGGTTGCGAGCACCT) previously described [42] was used and yielded a specific 162 bp long product. Before utilization, the specificity of the primers was tested and confirmed.

The amplifications were performed in 15 µL final volume containing 7.5 µL of Absolute Blue qPCR SYBR green low ROX mix (Thermo Fisher Scientific), 1.4 µL of primers mix (280 nM), 1.1 µL of DNase/RNase-free water and 5 µL template cDNA (10-fold diluted).

The PCR program was the following: an initial denaturation of 95 °C for 15 s followed by 40 cycles of 95 °C for 10 s, 60 °C for 45 s with a single fluorescence measurement before an elongation step of 72 °C for 30 s. The specificity of the PCR products was confirmed by melting curve analysis (after a step at 95 °C for 15 s, the melting curve analysis was performed between 60 and 95 °C with a heating rate of 0.1 °C/s). Different non-template controls and positive controls (with genomic DNA of *T. xylanilyticus*) were also included to confirm the specificity of the reactions. The *tx-xyn11* transcripts were normalized by using the expression of *T. xylanilyticus* 16 S rRNA gene amplified by using the specific primers (forward primer: CGCGAGCGACGCAATCCCA, reverse primer: CGGTTACCCCACCGGCTTCG). For the relative expression of *tx-xyn11* gene, calculation was done using the 2^−∆∆Ct^ method [43]. For the quantification of *tx-xyn11* transcripts over generations, the expressions of *tx-xyn11* at the beginning of the successive cultivations (generation 0) were used as reference samples. In order to compare, the expression of *tx-xyn11* gene between glucose and xylan successive cultivations, the reference sample was defined as the first cultivation on glucose. The results were expressed as the fold increase of mRNA level over the reference samples. Quantitative PCR was realized in triplicates for each sample of successive cultivations.

### Population analyses by flow cytometry

Population analyses at the different generations described above (for the successive cultivations) were done by flow cytometry with a BD Accuri™ C6 (BD Biosciences) using the MOBICYTE core facilities (University of Reims Champagne-Ardenne). The analytical parameters were flow rate of 35 µL/minute, core size of 16 μm and the threshold were down to 30,000 in FSC-H. 20 000 cells were collected for each sample.

Samples taken at different generations (5 mL) were filtered with 5 μm cellulose syringe filters and diluted with PBS 1× to reach maximum 2500 detected events/s (and events/µL) to prevent doublet reading. Samples were analyzed first unstained, and populations were detected by the forward and scatter signals (respectively FSC-A and SSC-A) to determine cell percentage of each detected population with a gating at 30 000 in FSC-A. The BD CSampler Software was used to acquire and treat the cytometric data.

Samples were also analyzed after staining with BacLight™ RedoxSensor™ Green (RSG, 1 µM) or propidium iodide (PI, 10 mg/L) (Invitrogen) to detect the metabolic activity and the membrane permeability respectively [37, 44]. After 488 nm laser excitation, green fluorescence were collected at 525 nm ± 30 nm for RSG (FL1-A) and the red fluorescence signal at 670LP (FL3-A) for PI.

Cells were also analyzed at different growth phases (exponential phase, stationary phase and sporulation) on different carbon sources (glucose, xylan and destarched wheat bran) with the same cytometric parameters.

### Cell sorting

The cell sorting was realized from *T. xylanilyticus* cultivated on xylan basal medium at the generation 0 but also along generations (G20 and G50). The cell sorting was performed with a BD FACSAria™ II Cell Sorter coupled with the BD Accuri™ C6 Plus flow cytometer from the URCACyt technical platform facilities.

In order to standardize the signals of the BD Accuri™ C6 Plus flow cytometer and BD FACSAria™ II cell sorter, an analysis of 2.5 μm microbeads (BD Biosciences) was done with both systems.

5 × 10^6^ events were collected for each population and the rate of sorting was around 2500 events/s with a 70 μm nozzle. Each cell sorting was performed in triplicate. The events were collected in 10 mL of PBS 1×. To check for a correct cell sorting, an analysis of each population collected was done with BD Accuri™ C6 plus flow cytometer. Each population solution was then centrifuged at 12,108×*g* (Sorvall ST 8R centrifuge, Thermo Scientific) for 30 min at room temperature. The cell pellet conserved in 100 µL of 1× PBS was solubilized with 1 mL of xylan basal medium. All the cell solution was used to inoculate a new cultivation on xylan and characterize the separated populations.

### Cultivations of the sorted populations

Population cultivations were performed as described in the previous parts for 19 h. The growth was followed with regular measurement of the OD_600 nm_. At the different growth phases determined (lag, beginning and end of the exponential phase and stationary phase), samples of 5 mL were taken and centrifuged at 3354×*g* for 10 min (Sorvall ST 8R centrifuge, Thermo Scientific). The supernatants were used for the measurements of xylanase activity and protein contents according to the procedures described above.

### Scanning electron microscopy analyses

During the followed successive cultivations, samples were taken before and after the cell sorting for analyses. Before the cell sorting, drops of 20 µL were dried on glass coverslip. After the cell sorting, 10 mL of different sorted populations (at 500 cells by µL corresponding to 5 × 10^6^) were concentrated by centrifugation at 16,000×*g* for 40 min. The cell pellets were solubilized in 500 µL and 22.5 µL drops were dried on glass slide (corresponding to 225,000 cells).

After drying, glass slides were washed 2-times in 1× PBS, then fixed in 2.5% (W/V in PBS) glutaraldehyde (Sigma-Aldrich) at room temperature for 1 h. After 2 distilled water washing, cells were dehydrated at room temperature in graded ethanol solutions (50, 70, 90, and twice with absolute ethanol) for 10 min and in a solution of ethanol (100%)/hexamethyldisilazane (V/V) for 5 min. Glass slides were finally desiccated with one final drop of HMDS. After air-drying at room temperature, samples were sputtered with a thin gold–palladium film using a JEOL ion sputter JFC 1100 instrument. Samples were then observed using a Schottky Field Emission Scanning Electron Microscope (JEOL JSM-7900 F).

### Successive cultivation on glucose

Successive cultivations were also performed by using glucose as carbon source during 80 generations. For the generations 23.9, 42.7, 62.9 and 79.9 (due to a different growth rate than on xylan), a switch of the carbon source between glucose and xylan was performed and the same analyses (flow cytometry and xylanase activity) as those for xylan successive cultures were done.

### Statistical tests

The different results of growth, xylanase activities, cell percentage and *tx-xyn11* gene expression were compared for the different generations by statistical analysis with Student’s test from MATLAB statistics and machine learning toolbox. Differences were considered significant for p ≤ 0.05. The same statistical analyses were performed to compare the two subpopulations after cell sorting.

## Supplementary Information

**Additional file 1: Figure S1.** Comparison of *tx-xyn11* gene expression level between cultivations on glucose and xylan.

**Additional file 2: Table S1.** Evolution of the relative growth rate and relative xylanase activity along generations.

**Additional file 3: Table S2.** Evolution of the growth rate on glucose along generations and xylanase activity after carbon source switch to xylan.

## Data Availability

The datasets used and/or analyzed during the current study are available from the corresponding author on reasonable request.

## Abbreviations

FC: Flow cytometry
FSC: Forward scatter
GH: Glycoside hydrolase
HMDS: Hexamethyldisilazane
IU: International unit
OD: Optical density
PBS: Phosphate buffer solution
PCR: Polymerase chain reaction
PI: Prodipidum iodide
RSG: RedoxSensor Green
SEM: Scanning electron microscopy
SSC: Side scatter
