# Supplementary figures and images for: Xylanase production by Thermobacillus xylanilyticus is impaired by population diversification but can be mitigated based on the management of cheating behavior
Source: Microb Cell Fact. 2022 Mar 15;21:39. doi: 10.1186/s12934-022-01762-z (PMC8922903; doi:10.1186/s12934-022-01762-z)

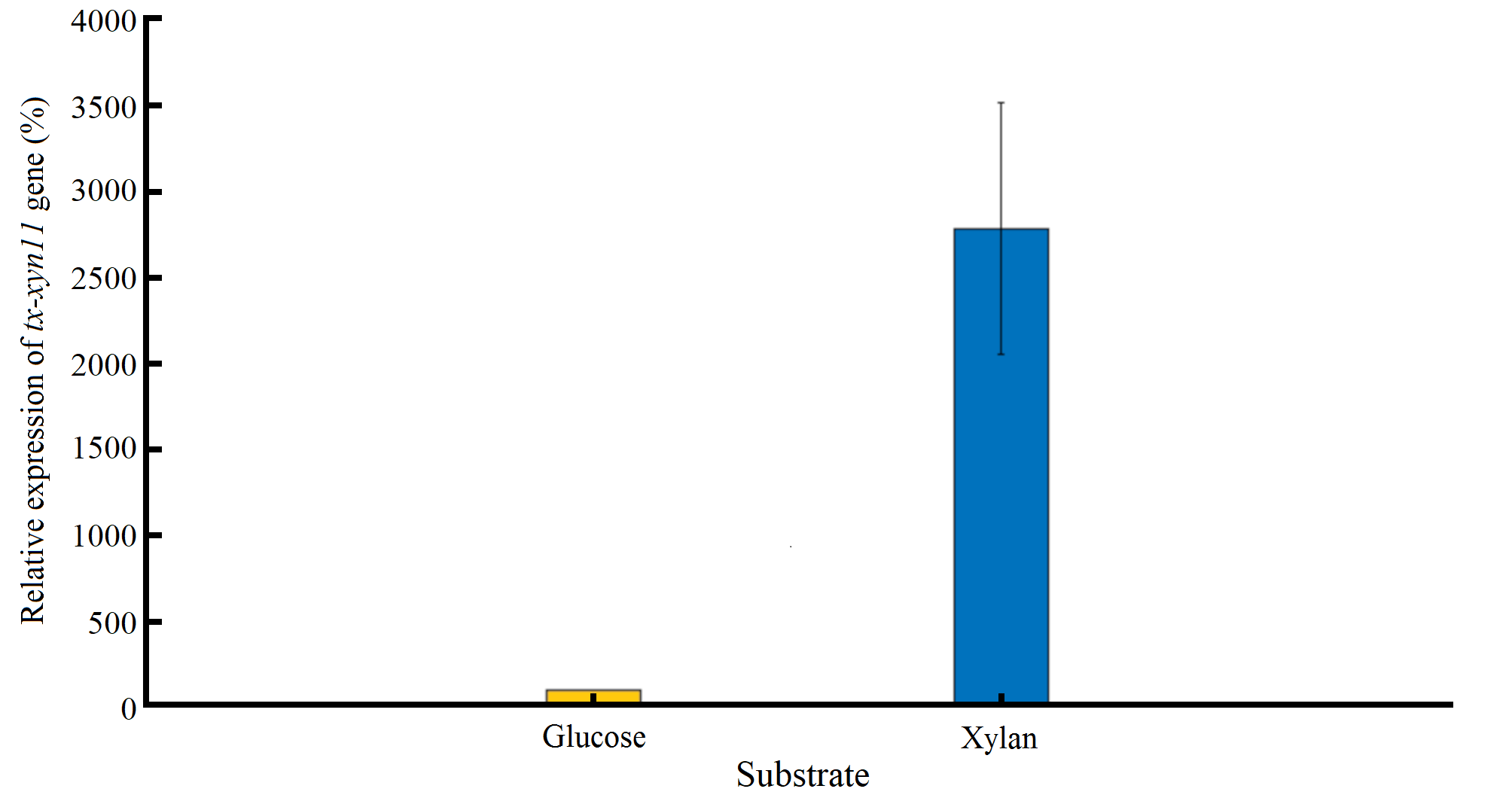

Supplement: Supplementary file 1 — Additional file 1: Figure S1. Comparison of tx-xyn11 gene expression level between cultivations on glucose and xylan. [file 12934_2022_1762_MOESM1_ESM.tiff]
