# Supplementary material for: Xylanase production by Thermobacillus xylanilyticus is impaired by population diversification but can be mitigated based on the management of cheating behavior
Source: Microb Cell Fact. 2022 Mar 15;21:39. doi: 10.1186/s12934-022-01762-z (PMC8922903; doi:10.1186/s12934-022-01762-z)
Supplement: Supplementary file 2 — Additional file 2: Table S1. Evolution of the relative growth rate and relative xylanase activity along generations. [file 12934_2022_1762_MOESM2_ESM.docx]

**Table S1:** **Evolution of the relative growth rate and relative xylanase activity along generations.**

| Number of generations | Xylanase enzymatic activity (IU/mg) – Mean ± SD | Relative* xylanase enzymatic activity (%) | Maximal growth rate (h^-1^) –  Mean ± SD | | Relative* maximal growth rate (%) |
| --- | --- | --- | --- | --- | --- |
| 0 | 141.36 ± 7.77 | 100 | 0.72 ± 0.12 | 100 | |
| 23.5 | 19.99 ± 4.60 | 14 | 0.95 ± 0.08 | 132 | |
| 50.8 | 35.40 ± 10.92 | 25 | 1.02 ± 0.05 | 142 | |
| 80.3 | 38.51 ± 11.78 | 27 | 0.97 ± 0.03 | 134 | |
| 107.7 | 18.51 ± 4.54 | 13 | 0.96 ± 0.06 | 133 | |

* The relative values were calculated by reporting the means values of a specific generation by the means values at the beginning of the cultivations and expressed as a percentage of activity or growth rate.
