# Supplementary material for: Xylanase production by Thermobacillus xylanilyticus is impaired by population diversification but can be mitigated based on the management of cheating behavior
Source: Microb Cell Fact. 2022 Mar 15;21:39. doi: 10.1186/s12934-022-01762-z (PMC8922903; doi:10.1186/s12934-022-01762-z)
Supplement: Supplementary file 3 — Additional file 3: Table S2. Evolution of the growth rate on glucose along generations and xylanase activity after carbon source switch to xylan. [file 12934_2022_1762_MOESM3_ESM.docx]

**Table S2:** **Evolution of the growth rate on glucose along generations and xylanase activity after carbon source switch to xylan.**

| Number of generations | Xylanase enzymatic activity (IU/mg) – Mean ± SD | Relative* xylanase enzymatic activity (%) | Maximal growth rate (h^-1^) –  Mean ± SD | | Relative* maximal growth rate (%) |
| --- | --- | --- | --- | --- | --- |
| 0 | 217.07 ± 84.20 | 100 | 0.87 ± 0.05 | 100 | |
| 23.9 | 196.48 ± 38.31 | 91 | 0.63 ± 0.05 | 72 | |
| 42.7 | 346.99 ± 47.07 | 160 | 0.68 ± 0.11 | 78 | |
| 62.9 | 260.62 ± 56.87 | 120 | 0.61 ± 0.14 | 69 | |
| 79.9 | 288.55 ± 10.70 | 133 | 0.80 ± 0.14 | 91 | |

* The relative values were calculated by reporting the means values of a specific generation by the means values at the beginning of the cultivations and expressed as a percentage of activity or growth rate.
